# The Gambler’s Fallacy: A Basic Inhibitory Process?

**DOI:** 10.3389/fpsyg.2013.00072

**Published:** 2013-02-20

**Authors:** James Lyons, Daniel J. Weeks, Digby Elliott

**Affiliations:** ^1^Department of Kinesiology, McMaster UniversityHamilton, ON, Canada; ^2^Department of Psychology, University of LethbridgeLethbridge, AB, Canada; ^3^School of Sport and Exercise Sciences, Liverpool John Moores UniversityLiverpool, UK

**Keywords:** attention, inhibition of return, gambling, illusion of control, gambler’s fallacy

## Abstract

Two studies were conducted to examine the relation between the gambler’s fallacy (GF) and attentional processes associated with inhibition of return (IOR). In Study 1, participants completed rapid aiming movements to equally probable targets presented to the left and right. They also completed a gambling protocol in which they bet on the illumination of either target. Consistent with the IOR phenomenon, participants were slower to initiate their movements on trial *N* + 1 when the target was the same as trial *N*. Participants with more pronounced IOR were more likely to switch betting behavior after a win than participants with a smaller index. This betting behavior was also related to a GF index measured by a questionnaire. In Study 2, participants performed both the aiming task and the betting task with a partner. Each participant performed two trials before ceding to the partner. Thus we were able to examine IOR and betting behavior as a function of the participant’s own previous trial and their partner’s previous trial. The IOR effect was robust both within and between-participants. Participants were more likely to maintain their bet following an unsuccessful outcome regardless of whether it was their own outcome or their partner’s outcome. This type of betting behavior is consistent with the GF. Individual IOR scores were a reliable predictor of betting behavior and the questionnaire was also successful in predicting behavior. In addition, the within-person IOR indices covaried with the GF index derived from the questionnaire. In summary, there appears to be a relation between IOR and the GF. We suggest that early humans developed specialized attentional systems to deal with non-random environmental contingencies, and that the automatic processes associated with these systems are sometimes maladaptive in artificial environments in which the same contingencies do not hold.

## Introduction

Over 50 years ago Heider (1958) suggested that one of the most important prevailing characteristics of human nature is a strong motivation to exert individual control over a personal environment. In most cases, this ambition of control is considered a positive human trait and is even cited as desirable in the fostering of mental health (e.g., Taylor and Brown, 1988). Of course, not all elements of life are subject to personal control. One example of this occurs when random events govern outcome, as is the case in games of chance. An illustration of this is seen in the oft cited observation that dice players throw the dice harder if they desire high numbers and vice versa (e.g., Henslin, 1967; Langer, 1975). Although a search of the literature has yet to uncover any empirical data to support this observation, the effect is considered to be quite robust, suggesting an innate desire to maintain a degree of control over a random outcome. This desire to exert control over external events is not limited to movements. Often, this desire extends to the perception of one’s ability to simply *predict* future random events. This desire is fundamental to many gambling behaviors.

All gambling behavior, at its most rudimentary, can be expressed as a sequence of actions based upon a series of decisions. By definition, an initial “bet or don’t bet” decision invariably entails the prediction of future events, with the confidence of these predictions serving as a significant mitigating factor in wagering behavior. At times, this predictive confidence is shaped on the basis of a rational assessment of outcome probability and an evaluation of the principles of randomness. For example, if a person draws an Ace from a deck of 52 cards and then sets it aside, the probability of drawing an Ace on any subsequent attempt is lower (0.057 vs. 0.077). In this case, where the outcome of the second trial is contingent upon the outcome of the first, it is wholly appropriate for the predictive confidence in drawing a second Ace to be lowered. If, on the other hand, the first Ace is replaced in the deck the probability of drawing an Ace again does not change (i.e., the two events are not contingent). Thus, a problem is created when the bettor assumes a level confidence that is not warranted because non-contingent probabilities are assessed in a similar manner to contingent probabilities. This predictive behavior has been described as coming under the broad descriptor of the Illusion of Control (Presson and Benassi, 1996) and represents a specific instance of this illusion: the Gambler’s Fallacy (GF).

The GF generally refers to the misconception that the probability of occurrence of an event or outcome is influenced by a previous, or series of previous, events. More specifically, the Fallacy is typically manifest by the following beliefs: a random event is more likely to occur because it has not happened for a period of time; and a random event is less likely to occur because it recently happened. Thus events, are believed to be related, even though the probabilities associated with their occurrence is objectively known to be independent. The GF is a powerful and seductive illusion of control over events that are, by definition, not controllable^1^.

Support for such an inherent susceptibility to illusory control is found in evidence that individuals can be *induced* to believe that they can affect the outcome of purely random events. Participants who correctly guessed a series of coin tosses began to believe that they were, in fact, better at predicting outcomes than were others. This confidence in success, often termed high “self-efficacy” can be particularly problematic in gambling situations since there is considerable empirical evidence to suggest that high levels of self-efficacy can be maladaptive. Whyte et al. (1997) showed that participants in whom they had induced high self-efficacy were significantly more likely to escalate commitment to a failing course of action. It should not be surprising then that such types of spurious assumptions have been suggested to be a significant contributing factor to the maintenance of gambling behavior (e.g., Benhsain et al., 2004).

In summary, The GF (and more broadly defined, Illusion of Control) is considered to be an important determinant of decision-making in gambling behavior. Traditionally, the processes that give rise to the Fallacy have been thought to occur at a fairly high level of cognitive processing. As such, previous investigations have explored issues of working memory (Kareev, 1995), overt awareness of independence (Benhsain et al., 2004), signal detection (Erev, 1998), stochastic models of the perception of randomness (Nickerson, 2002), and even principles of Gestalt grouping (Roney and Trick, 2003). However, a possibility is that the locus of the Fallacy might in fact originate at a much lower level. This reasoning stems from research showing that individuals demonstrate a tendency to respond more slowly to a repeated event than a new event, even though the probability of these two events is equal. This highly reproducible effect is thought to result from a very basic inhibitory process in the central nervous system that has evolved to facilitate efficient search behaviors. The phenomenon has been termed inhibition of return (IOR).

Imagine a person picking apples in an orchard. After picking all of the apples from Tree A, is her original goal of maximizing apple accumulation best served by returning to Tree A, or by moving on to Tree B? Although this example might be considered obvious (or even facetious) it is often used to illustrate a relatively complex neurobiological phenomenon that has come to be described as IOR. Originally revealed and studied in some detail almost 30 years ago by Posner and Cohen (1984), IOR refers to a phenomenon in which response times are delayed when those responses are based upon stimulus events originating from a previously cued object or location. The reasons behind the existence of IOR effects remain unclear although it has been suggested that the effect is an evolutionary adaptation, originating in lower-order neural processes, that serves to prevent the return of an organism to a previously explored, and presumably now inadequate, location in space^2^.

A major goal of research over the past 15 years has been to uncover the origins of IOR by examining, amongst other things, the phenomenon relative to intrinsic (e.g., location, shape, color, predictability, etc.), and extrinsic (e.g., sensory modality, dynamic vs. static display, etc.) stimulus characteristics, and the planning and execution of target-directed ocular movements (Taylor and Klein, 1998). Although such previously cued responses may often be facilitated if the cue and target are separated by a brief interval (i.e., 100–200 ms for visual stimuli; Posner and Cohen, 1984), responses to the cued target are reliably slowed if target onset occurs more than 300 ms after the presentation of the preceding cue (Tassinari et al., 1994; Schmidt, 1996; Danziger and Kingstone, 1999; Briand et al., 2000; Schmitt et al., 2000).

Although originally studied in situations in which a target follows a non-informative cue (i.e., the cue-target paradigm), there is also a robust IOR effect in serial target-aiming situations. In this so-called “target–target” paradigm (Maylor and Hockey, 1985), the initiation time for aiming movements on trial *N* + 1 is longer if the movement must be made to the same target as on trial *N*, rather than a new target location (e.g., Tremblay et al., 2005). This target–target IOR effect occurs even when the interval between consecutive trials is several seconds (Tremblay et al., 2005). Facilitation is not a factor in the target–target paradigm, because the interval between consecutive aiming movements is always >300 ms and usually in the order of several seconds.

It has been hypothesized that the initial facilitation period, followed by IOR, has evolved as an attentional mechanism to allow humans to effectively search their environment (Posner and Cohen, 1984). In other words, it is usually advantageous to inhibit already inspected and/or engaged targets so that more attentional resources can be directed toward novel stimuli/targets.

The purpose of the two studies reported here was to determine whether or not there is any relation between the expression of the GF in betting behavior and the basic attentional process of IOR. Our notion was that both the GF and IOR stem from the same low-level neural processes. These processes are generally adaptive in the real world where environmental contingencies are non-random. However, in the random environment of a casino or experimental psychology laboratory they result in biased (GF) and slowed (IOR) decision-making respectively.

## Study 1

### Method

#### Participants

Thirty adults from the McMaster University community participated in both tasks. Half of the volunteers were male. All participants were right-handed, between 18 and 26 years of age and naïve to the purpose of the experiment. Participants were remunerated $10 for their time. Before beginning the experiment, each participant provided written informed consent to participate in the experiment. The procedures were approved by the McMaster University Research Ethics Board.

#### Apparatus and tasks

Participants sat in front of a table that held the aiming apparatus. This apparatus consisted of a metal board (43.5 cm × 33.5 cm) containing three illuminating button-switches (1.4 cm in diameter). At the center and near edge of the board, one button-switch served as a home position for the index finger of the right hand. The two peripheral button-switches (14 cm in front of and 14 cm to the left and right of the home position) could be illuminated to indicate the target location for a given trial. A cross was positioned between the target positions and served as a central fixation marker. E-Prime™ version 1.1a was used to randomly display target stimuli and to record the timing and the order of button release and contact.

Participants also filled out a questionnaire that we developed to examine susceptibility to the GF (see Appendix). The questionnaire consisted of 15 statements about gambling, and participants were asked to indicate how strongly they agreed/disagreed with each statement on a seven-point Likert scale. Items 3, 4, 6, 8, 10, 11, and 12 were designed to be GF items with reverse scoring on item 10. The other items were fillers. The GF items were used to calculate a GF Index. A check of the overall internal consistency of the seven-GF items yielded a Cronbach’s Alpha of 0.904. Individual correlational analyses of these items revealed that individual GF scores in Study 1 were moderately and highly related to each other (median *r* = 0.584; 25th‰ = 0.514 and 75th‰ = 0.689). The relation between Gambler Fallacy scores and other item scores was modest or absent (median *r* = 0.1765; 25th‰ = 0.0855 and 75th% = 0.3465). An analysis of the *z*-transform of these correlations coefficients revealed that the difference was highly significant, *t*(75) = 8.41, *p* < 0.0001 (GF items with each other: *z* = 0.709; GF items with other items: *z* = 0.237).

The GF scores were tallied, after correcting for the reverse item, to create a GF Index. Although based on the total of seven-GF Likert items, the distribution of GF Indices across the 30 participants approached normality (e.g., skewness = 0.007), and thus could be used in parametric analyses to predict outcomes associated with our two main experimental protocols.

#### Procedure

For the first task, participants were instructed to maintain fixation on the central cross throughout the block of trials. The target location for each trial was indicated by a 100 ms illumination of one of the peripheral buttons. During each series of movements, the participant was instructed to begin with the index finger of their right (dominant) hand on the start position and to move their finger as fast and accurately as possible to the illuminated target button. Once the participant had depressed the target switch, 1000 ms elapsed allowing him or her to return “home” and prepare for the next trial. Thus along with the reaction time (RT) and movement time (MT), the inter-trial internal was approximately 1550–1600 ms.

The location of the target for each trial was random with the following constraints: (1) left and right locations were presented as the target an equal number of times across the entire experiment; and (2) no target appeared at one location more than *five* times in a row. The records produced by E-Prime were used to determine: (1) if the participants moved to the correct target location; and (2) the time elapsed from target onset until home position release (RT) and the time elapsed from home position release until target button contact (MT). For this task participants completed four blocks of 101 successive rapid aiming movements to target stimuli that appeared at one of two locations. Participants were given a brief rest between trial blocks.

The second task used the same apparatus as the first. However, for this task, participants were asked to predict which peripheral button (left or right) would illuminate and to place a bet based on this prediction. Each participant was given $2 in nickels to use for betting purposes. Participants were informed they could place a bet of 5 cents for each bet and that if their bet was correct they would win an additional 5 cents, but if the bet were incorrect, they would lose the 5 cents they placed as a bet. Participants were also informed that they would get to keep any winnings they made over the $2 starting budget. Each participant started by pressing one of the peripheral buttons and placed a nickel beside this button. After the button press, the target illuminated and the experimenter, depending upon the outcome, took away the nickel or gave the participant an additional nickel. This protocol continued until the block of trials was completed. The location of the target for each trial followed the same constraints as the aiming protocol. The records produced by E-prime were used to determine location of the bet as well as the outcome of the bet. Each participant completed four blocks of 101 successive bets. After completing both protocols participants were asked to fill out the questionnaire described above.

Before both the IOR protocol and the gambling protocol, participants were told that the light illuminated on each trial was completely random.

#### Data analysis

For the first task, we analyzed RT and MT using separate 2 (previous target: same, different) × 2 (target location: left, right) repeated measures analysis of variance to verify that we replicated previous findings of IOR (e.g., Lyons et al., 2006). We discarded the first trial of each block because there was no previous trial with which to refer. We removed any trial that did not fall within two standard deviations of the mean for a particular participant, and then recalculated the means. A total of 3% of all trials were removed from the analysis. No aiming errors were made in Study 1.

To determine if IOR was related to betting behavior associated with the second protocol, we conducted a series of correlational analyses to examine the relationship between the strength of individual participant’s IOR effect and their betting behavior. An IOR index was calculated for each participant. The IOR index was calculated as follows: (RT same mean − RT different mean)/RT grand mean for that particular participant. This calculation was done separately for the left and right locations and the two indices were averaged for an overall index. For betting behavior, we determined the proportion of total bets placed in each location (same or different than last bet) according to the outcome of the previous bet (won or lost). Thus, we had a measure of proportion bets based on 4 different events: (1) a bet placed in the same location as the previous bet when the previous bet was won or (2) when it was lost (3) a bet placed in a different location than the previous bet when the previous bet was won or (4) when it was lost. These proportions were based on a total of 400 trials for each participant, except one participant who failed to respond on 1 of the 400 trials. The first trial in each block was discarded because there was no previous trial with which to refer.

In addition to analyzing the correlation between the IOR index and betting behavior, we also included each participant’s GF score in the analysis. This score was obtained from the questionnaire and based on the sum of responses to seven questions that were answered on a seven-point Likert scale. Higher numbers indicate a stronger tendency to have misconceptions about chance events that are typical with the GF.

### Results

#### Aiming results

**Figure 1:**
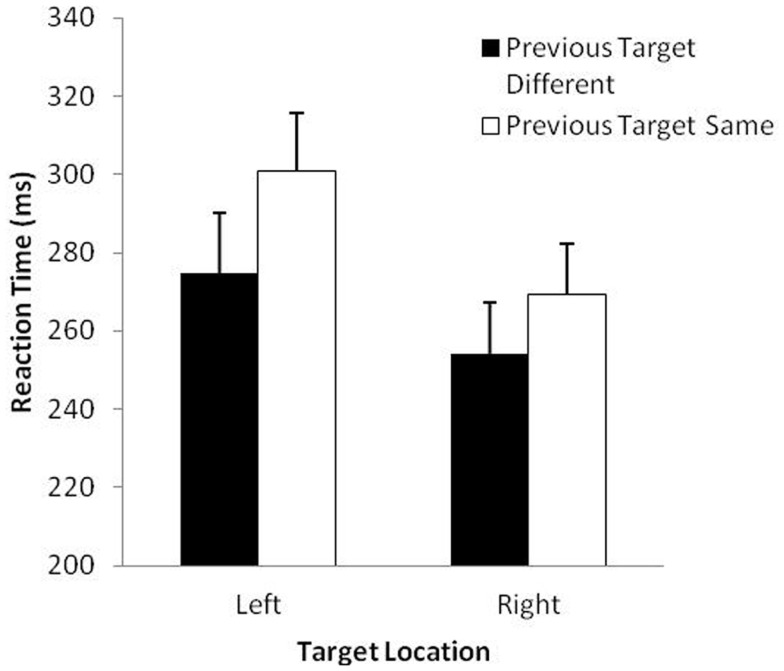
**Mean reaction time (ms) and standard error (bars) as a function of previous target and target location in Study 1**.

The analysis of RT revealed significant main effects of previous target, *F*(1, 29) = 67.2, *p* < 0.001 and target location, *F*(1, 29) = 66.3, *p* < 0.001. Overall, participants took longer to prepare movements to the same target (285 ms) than to different targets (264 ms) and took longer to prepare for movements to the left (288 ms) than the right (262 ms). These effects were modulated by a significant Previous Target × Target Location interaction, *F*(1, 29) = 23.4, *p* < 0.001 (see Figure 1). The difference in RT for same vs. different previous target was slightly larger when the location of the target was on the left rather than the right. As is typical in most IOR studies, the MT analysis revealed only a main effect for side, *F*(1, 29) = 23.49, *p* < 0.001, with participants executing aims to the right-sided target (289 ms) slightly faster than to the left-sided target (323 ms). This finding was not surprising because all participants were aiming with their right hand (see Elliott et al., 1995).

#### Betting results

The results of the gambling behaviors and outcomes were straight-forward. Overall participants actually won (51.7%) on slightly more than they lost (48.3%), *F*(1, 29) = 5.34, *p* < 0.05. However neither the percent win nor percent lose scores was significantly different from the expected value of 50%, *p* > 0.05. Interestingly, participants more often maintained (57.4%) than switched (42.6%) their betting behavior from trial-to-trial, *F*(1, 29) = 12.98, *p* < 0.01. This tendency to stay with their previous bet occurred regardless of whether they won (58.2%) or lost (56.8%).

#### Correlational results

Although the IOR index did not predict what participants did after a loss [i.e., stay after a loss: *r*(28) = 0.097, ns], it did predict what participants did after a win. Specifically, the IOR index was positively correlated with the proportion of trials in which participants switched bet locations following a win [*r*(28) = 0.328, *p* < 0.05; *z* = 0.341 with a 0.90 confidence interval of 0.031–0.651]. Thus people who were more influenced in their aiming behavior by a previous trial were also more likely to shift their betting behavior to the other target after a win.

The analysis of correlation between the GF indices measured using the questionnaire and betting behavior also revealed some significant relationships. Specifically, the GF index was positively correlated to the percentage of trials in which participants switched bet locations after a loss [*r*(28) = 0.352, *p* < 0.05, one-tailed; *z* = 0.369 with a 0.90 confidence interval of 0.059–0.679]. Thus people who are more likely to cognitively subscribe to the GF also tend to shift their betting behavior following a loss. Interestingly there was very little relationship between the GF index and the IOR index [*r*(28) = 0.11, ns].

### Discussion

Study 1 was conducted to determine whether the behaviors associated with the expression of the GF are consistent with those observed with the IOR phenomenon. Thus, data were collected with respect to individual participants’ performance across two distinct protocols: a two-choice betting paradigm and a two-location aiming task. In addition to these two laboratory tasks, prospective data were also collected probing each individual’s attitude toward perceptions of chance events in general and attitudes toward gambling behaviors in particular. These data were then used to construct individualized GF matrices (e.g., a measure of susceptibility of GF beliefs). Our primary hypothesis was that a reliable associative relationship exists between an individual’s susceptibility to the IOR effect (as determined by individualized IOR indices) and the manner in which they bet. Specifically, it was predicted that the more susceptible a person was to IOR, the more likely it would be that they would change the location of subsequent bets following a win and re-bet the same location after loss.

Although the results of this study revealed a generalized IOR effect (RTs were longer for movements to the same target than they were for movements to a different target), they were inconsistent with our primary hypothesis. Specifically, analyses of the betting behaviors revealed that participants were reliably more likely to re-bet to the same location rather than switch location. This tendency to maintain the location of a previous bet was maintained whether that previous bet resulted in a win or a loss.

When individual differences are considered, however, a somewhat different picture emerges from that of the group data. For example, the correlational results examining the relation between individualized IOR aiming indices and trial-to-trial bet locations revealed that individuals whose aiming behaviors demonstrated a greater susceptibility to IOR were more likely to switch bet locations after a win. Furthermore, when these individual betting behaviors are examined in the context of personal propensities toward the GF, there was evidence that these two variables are also related. In particular, a significant relationship between GF indices and bet location was revealed suggesting that the greater the degree to which a person subscribes to GF beliefs, the more likely they are to switch the location of bets that follow losses.

Taken together, the results of Study 1 provide putative evidence that the GF and IOR effects are subserved by similar underlying mechanisms. The data from this study do not, however, support the position that this is a general effect. Rather, the results from Study 1 suggest that the degree to which the GF and IOR effects are related is more complex and may depend on situational variables as well as individual differences. Study 2 was conducted to replicate Study 1 (i.e., as a guard against any Type 1 errors), and extend our investigation of the GF and IOR phenomena to situations in which people perform with a partner.

## Study 2

Gambling is a social behavior. By way of illustration, a comprehensive study of adolescent gambling behaviors by Gupta and Derevensky (1998) revealed that of 665 respondents who acknowledged involvement in gambling, 76% reported that they typically engaged in these behaviors with friends, family, and/or strangers. Indeed, there are now several decades of research suggesting that any rational assessments of “gambling behaviors” are only meaningful when considered in the social and cultural contexts in which they occur (e.g., Abt et al., 1985). For example, evidence from Rockloff and Dyer (2007) suggests that the presence of other players, particularly when those players appear to be winning, can have a direct effect on risk taking behaviors. In this study, when participants received feedback information (e.g., winning bells, instant messages, etc.) regarding the wins of other players, they placed a greater number of bets, and lost more money. Furthermore, recent evidence (Eiji Nawa et al., 2008) suggests that the presence of others in these types of bet-to-win scenarios result, not only in direct changes to betting behaviors, but also in significant modulation of areas of the neural architecture thought to be involved in motivation (i.e., amygdala, medial prefrontal cortex, and striatum).

Generally, studies that have examined the presence of others as a mediating influence in gambling behaviors have framed the issue in terms social facilitation (e.g., Abt et al., 1985; Gupta and Derevensky, 1998; Rockloff and Dyer, 2007). There is, however, another way to approach the question. In certain games of chance, the ways in which a player adapts his/her betting behavior is often directly dependent, not only upon the presence of other players, but also upon the specific *actions* of those other players and the outcomes associated with those behaviors (e.g., Blackjack, Craps, etc.). Thus, it is possible to explore not only the effects of social facilitation on these behaviors, but also the effects of social affordance. In this context, social affordances simply refer to the properties of constituent components within an environment (including other people) that permit or prime social actions and/or interactions (Gaver, 1996).

In the context of a multi-person gambling experience, this “offering” of action possibilities can take several forms. At its most simplistic, if two people are cutting a deck of playing cards to determine who deals the next hand, the actions of the first player (cards taken) imposes specific action limits upon the second player (cards left). At a more complex level, if one player’s actions result in a string of consecutive losses on a particular slot machine (causing them to leave) a second player observing this may drastically modify his/her upcoming action plan (e.g., leave the slot machine they are playing and move into the vacated seat, wager a larger amount on that machine, etc.). Both of these scenarios constitute examples of social affordances in that the actions of one person either necessarily or potentially affect the action possibilities of the other. In the second scenario, it is important to note that these subsequent action possibilities are not simply affected by physical properties (e.g., cards left in the deck) but also by the perception of a changed environmental dynamic (i.e., a consecutive string of losses). That this perception is, in fact, a misperception returns the discussion to the GF.

**Figure 2:**
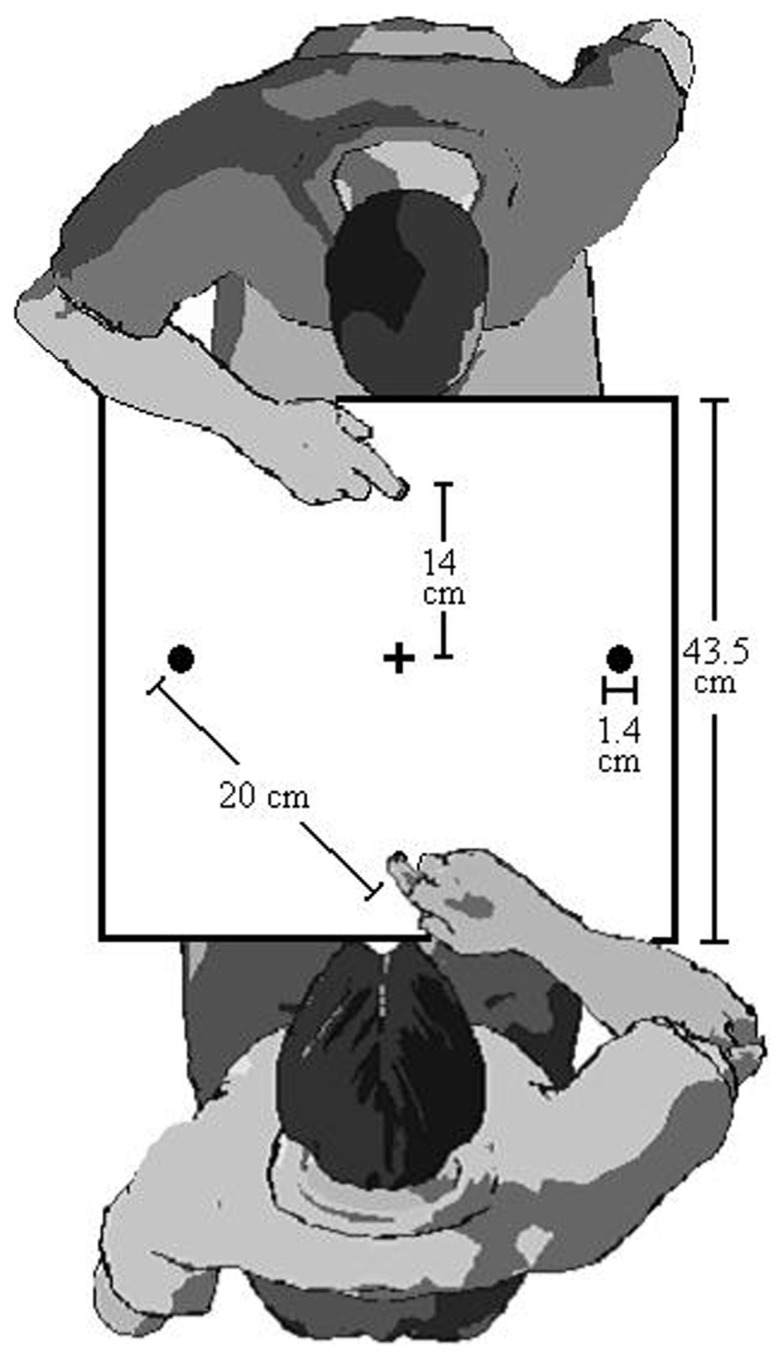
**Apparatus for IOR and betting protocol in Study 2 (In study 1, there was only one person)**.

We have previously demonstrated that between-person IOR exists. In what we call the “Joe and Fred” series of experiments, we had two participants sit across the table from each other as depicted in Figure 2. Joe and Fred alternate turns responding to the target lights. Each participant performs two consecutive trials before yielding to the other participant. This trial scenario makes it possible to examine Joe’s RTs and MTs following his own trials, and also after Fred’s trials. In our initial experiment (Welsh et al., 2005, Experiment 1) both a robust within- and between-person IOR effect was found. This between-person IOR effect persisted when we used liquid crystal goggles to eliminate vision of the co-performer’s target-illumination, but not his/her aiming trajectories (Welsh et al., 2005, Experiment 2). Moreover between-person IOR was evident when Joe was only able to see the result of Fred’s aiming movement (i.e., target contact, with no vision of the signal or trajectory) or one-third of the early movement trajectory (Welsh et al., 2007; Experiment 1 and 2A). When we used a white rectangular light to mimic the information about the early trajectory information provided in Experiment 2A, between-person IOR disappeared (Welsh et al., 2007, Experiment 2B). Together these effects lead us to suggest that between-person IOR is due to the mirror neuron system (Welsh et al., 2005, 2007; Hayes et al., 2010b).

The mirror neuron system consists of groups of neurons in the prefrontal and posterior parietal areas of the brain that become active during both the performance of an action and when observing another person’s actions (Di Pellegrino et al., 1992). Consistent with the notion that between-person IOR effects are associated with the mirror neuron system are findings that young adults with autism spectrum disorder (ASD) show within- but not between-person IOR effects (Welsh et al., 2009). People with ASD have been suggested to have dysfunctional mirror neuron systems (Iacoboni and Dapretto, 2006).

Given the findings of Welsh et al. (2005, 2007, 2009) and the results of Study 1, we designed Study 2 to address the question of how the presence and actions of others affects the degree to which the GF and IOR are related. To accomplish this, similar protocols to Study 1 were employed except that two people participated within the same experimental session. In order to keep the IOR protocol as similar as possible to the gambling protocol and real world gambling situations, we used the same procedures as Welsh et al., 2005, Experiment 1). Consistent with both the results of Welsh et al. (2005, 2007), as well as with the concept of social affordance, it was predicted that the presence of two betting partners would result in a strengthened degree of correlation between IOR and the manifestation of betting behaviors in keeping with the GF.

### Method

#### Participants

The participants were 20 right-handed young adults (10 females) between the ages of 19 and 25 years. Participants all provided informed consent and were paid $10. The procedures were approved by the McMaster University Research Ethics Board.

#### Apparatus and task setup

The target-aiming apparatus was identical to Study 1 except there were home positions on both sides of the board. This setup allowed a pair of participants to sit across from each other and see exactly the same target layout (see Figure 2).

#### Procedure

The target-aiming procedure was identical to that used by Welsh et al., 2005, Experiment 1). Participants sat across a table from each other with the index finger of the right hand on their own home position. The fixation, target-illumination, and stimulus timing constraints were the same as in Study 1. The difference was that the two participants took turns with the target-aiming. Specifically, each participant would perform two target-aiming trials and then concede the target-aiming board to his/her partner (e.g., P1, P1, P2, P2, P1, P1, P2,…). Thus on half the trials, each participant followed his/her own previous response and on the other half, his/her partner’s response. This protocol allowed us to examine both within-person and between-person IOR. Each pair of participants completed three blocks of 129 successive rapid aiming movements to one of the two target locations. A short break was given between each trial block. The experimental design allowed us to once again examine same and different trials in both left and right space and also whether consecutive trials occurred within or between individuals. Thus mean RT and MT were analyzed using separate 2 Person (within, between) × 2 Target Position (same, different)  × 2 Previous Location (left, right) repeated measures analyses of variance. We used the same outlier procedure as in Study 1, and eliminated less that 4% of all trials. Once again there were no aiming errors.

Similar to Study 1, we used the mean RT scores for each participant to calculate IOR indices. Although, we once again pooled right and left-sided indices in this study we calculated separate between and within-person indices. This procedure provided us with information about the strength of the inhibitory effect following one’s own response and also after watching another person respond.

The betting protocol used in this study was identical to the aiming protocol in that each participant made two bets before yielding the betting apparatus to their partner. This procedure allowed us to calculate 2 IOR indices.

### Results

#### Aiming results

**Figure 3:**
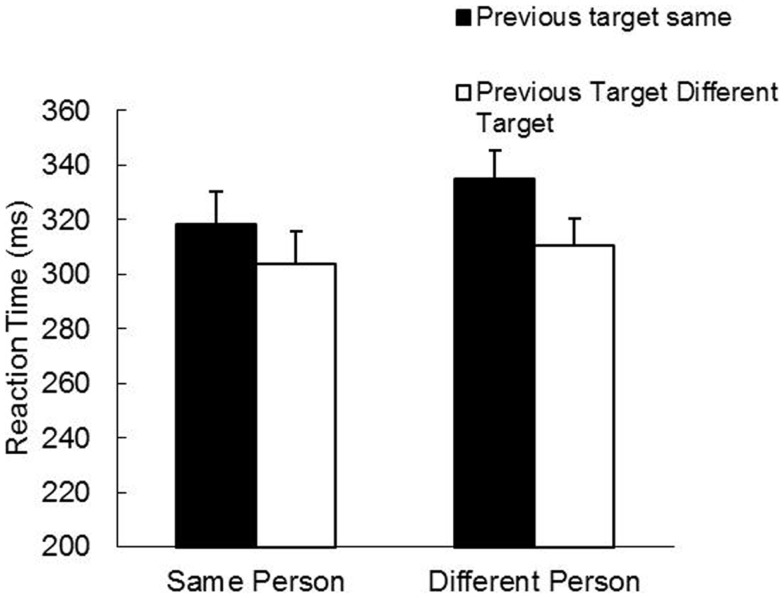
**Mean reaction time (ms) and standard error (bars) as a function of person and previous target in Study 2**.

The RT analyses revealed main effects for Person, *F*(1, 19) = 13.17, *p* < 0.01, and Target Position, *F*(1, 19) = 55.92, *p* < 0.001, as well as a Person by Target Position interaction, *F*(1, 19) = 6.25, *p* < 0.05. As is evident in Figure 3, there was an overall IOR effect and this effect was actually slightly stronger under between-participant conditions than within a participant (Between-Same: 335 ms; Between-Different: 310 ms; Within-Same: 318 ms; Within-Different: 304 ms). The MT analyses revealed no significant effects. The grand mean was 291 ms.

#### Betting results

In Study 2, participants won on 51.0% of their bets and lost on 49.0% of their bets. These percentages were not different from each other or the expected percentage of 50% (*p* > 0.20). When individual participants bet two times in a row, they were more likely to maintain their betting behavior (57.3%) than switch (42.7%). However, unlike Study 1, this tendency was mediated by outcome. Specifically, when participants were incorrect on the previous trial, they were more likely to maintain their betting behavior (65.9%) than when they were correct (49.6%), *F*(1, 19) = 11.22, *p* < 0.01. Thus participants switched their bets on 50.4% of the correct trials and 34.1% of the incorrect trials.

When a participant’s bet followed his partner’s bet there was not an overall tendency to maintain the betting behavior of the other individual (maintain = 47.1%, switch = 52.9%, *p* > 0.05). However betting behavior was influenced by the outcome of the partner’s bet. Specifically, when the partner was incorrect, participants were more likely to make exactly the same bet (55.6%) than when they were correct (39.0%), *F*(1, 19) = 11.74, *p* < 0.01. Thus participants switched their bets on 61% of correct trials and 44.4% of incorrect trials. From these results, it appears that the GF extends beyond a single gambler with observation impacting behavior similar to one’s own betting results.

#### Correlational results

In this study, we calculated separate within and between-person IOR indices. Interestingly, although the between-person IOR index was unrelated to betting behavior (*p* > 0.10), persons with a higher within-person index were more likely to switch their betting behavior when they were correct [*r*(18) = 0.52, *p* < 0.05, *z* = 0.576 with a 0.90 confidence interval of 0.120–0.920] than maintain the same bet. When following a partner, people with higher within-person IOR indices tended to bet on the same color light when their partner was incorrect [*r*(18) = 0.63, *p* < 0.05; *z* = 0.741 with a 0.90 confidence interval of 0.341–1.141]. Both of these betting patterns are consistent with the GF.

The GF index as calculated from the questionnaire was positively related to maintaining the same bet as a partner when the partner’s bet was wrong [*r*(18) = 0.50, *p* < 0.05; *z* = 0.549 with a 0.90 confidence interval of 0.149–0.949]. Again this behavior is consistent with the philosophy that the next result will be the reverse of the last result. The result supports the validity of questionnaire at least for between-person situations.

Finally, we examined whether or not there was any covariation between the questionnaire results and the IOR indices. Here, we found a moderate relationship between the within-person IOR index and the GF index [*r*(18) = 0.42, *p* < 0.05, one-tailed; *z* = 0.448 with a 0.90 confidence interval of 0.048–0.848]. The relationship between the GF index and the between-person IOR index was close to zero (*r* = −0.04).

### Discussion

This study was designed to extend our investigation of the relationship between the IOR phenomenon and the GF to a social context. As in Study 1, we were able to replicate the basic target–target IOR effect. As well, we again demonstrated that this effect is not confined to a single nervous system (e.g., Welsh et al., 2005, 2007; see also Hayes et al., 2010b). That is, participants were slower responding to the target that their partner had just engaged than the other target. This between-person IOR effect is consistent with the idea that the mirror neuron system is important in a number of areas that involve interpersonal interaction. These include motor skill acquisition via observation (Hayes et al., 2010a), language acquisition (Iacoboni and Dapretto, 2006) and, in the context of this experiment, the development and expression of social behaviors (Rizzolatti and Craighero, 2004). Also consistent with our interpretation of our current IOR data is the finding that between-person IOR effects are not present in persons with ASD (Welsh et al., 2009). These individuals are thought to have dysfunctional mirror neuron systems (Iacoboni and Dapretto, 2006). Thus, at least for most of us, “seeing” can have as large an impact on performance as actually “doing” (Welsh et al., 2007).

Like Study 1, our betting protocol revealed that participants were more likely to place the same bet two times in a row than change the bet. More interestingly, this behavior was most pronounced following a losing bet. As well, this tendency to follow a losing bet with the same bet was also present following the partner’s loss. Because the participant saw both the outcome of the previous trial and their partner’s bet however, the participants betting behavior could have been related to the target light defining the outcome, their partner’s successful/unsuccessful decision or both. In any case, both our within and between-person findings are consistent with the GF.

With respect to individual differences in betting behavior however, it was the within-person IOR index that did the best job of predicting betting. That is, following an incorrect bet participants with higher within-person IOR indices were more likely to maintain the same bet than switch. This result occurred regardless of whether the incorrect bet was his/her own bet or the partner’s bet. This betting behavior is consistent with the GF.

Interestingly, the within-person IOR index was the only index related to GF index measured by the questionnaire. A higher GF index was also related to participants maintaining a bet when the partner had just been unsuccessful with the bet. Thus in Study 2, we have support for the notion that IOR, betting behavior and outcome beliefs are interrelated.

## General Discussion

The overall purpose of this work was to determine if there was any relation between the GF and basic attentional inhibitory processes that may have evolved to facilitate search activities of early humans. In both studies we were able to demonstrate robust IOR effects, and in Study 2 the IOR effect was present both within and between nervous systems. As well, in both studies we found that the betting behavior of our participants was consistent with the GF. Specifically, participants were more likely to switch their betting choice after a win than after a loss. Of greater interest were the modest, but significant, relationships found between individual IOR indices and betting behavior. That is, people with higher IOR indices were more likely to switch their bets following a win and maintain their bets following a loss than participants with lower indices. The questionnaire results were also moderately related to gambling behavior and, in Study 2, susceptibility to the GF was also related to the within-person IOR index.

Although our findings are correlational, they are consistent with the notion that the GF and basic attentional orienting behaviors share some common information processing roots. If anything, the relationship between IOR and gambling behavior was strongest in a social setting (i.e., Study 2). Perhaps this is not surprising given that early hunting and food gathering activities, as well as most gambling behaviors, occurs in social environments (e.g., Gupta and Derevensky, 1998).

So how could a maladaptive approach to gambling, such as betting behaviors consistent with the GF, find its roots in an attentional phenomenon that may have been important for the early survival of our species? The answer may stem from the probabilities associated with particular events and outcomes in both the real world and the casino. In a casino, outcomes are designed to be random whereas in the real world this is usually not the case. For example, when we developed the evolutionary account of IOR using the apple-picking scenario, we postulated that apple pickers inhibit an area of visual space, or perhaps an action associated with a just-picked-apple, in order to turn their attention to new locations and new apples. In fact, the probability of a new apple being in the same location as a just-picked-apple is zero (at least until next year). That is, the probability of event *N* + 1 is contingent on event *N*. As individuals, and as a species, we learn about these real world probabilities by monitoring outcomes. Presumably we develop strategies and, over generations, cognitive and neural systems to optimize performance. The fact that these systems are common across individuals is evidenced behaviorally by the between-person IOR effects found here and elsewhere, and anatomically by the mirror neuron system that provides the foundation for these between system effects. The cognitive and neural systems that support performance in the real world cannot be expected to contribute to optimal outcomes in an artificial environment where the probabilities of one event are not contingent on previous events (i.e., the casino where outcomes are random).

Although the GF is just one example of an “illusion of control,” perhaps we can understand other misperceptions of control within a similar theoretical framework. Specifically, our survival as a species depended on early humans exercising control over their environment. In most cases, this approach to daily life is still adaptive. Consistent with theorizing about the origins of IOR, our ancestors developed some specialized neural processes to facilitate optimal outcomes. This would have been the case for the control of goal-directed action (e.g., Elliott et al., 2004) and more general perceptual decision-making (Posner and Cohen, 1984). Because at least some of these processes are automatic (e.g., IOR), they are at least temporarily immune to higher-order influences associated with a specific environmental context. Thus they operate not only in ecologically valid contexts, where they are adaptive, but also in artificial contexts such as a gambling and/or casino environment, where they have no impact on outcome. In the context of selective attention and goal-directed action, the IOR phenomenon is not a unique inhibitory process. For example, limb movements made to a target object often veer away from the straightest path in order to avoid contact with another (non-target object) if the object is close to the movement path (e.g., Welsh and Elliott, 2004). This avoidance behavior can be adaptive when the non-target object is real. However deviations in movement trajectories also occur to “accommodate” distractors on a computer screen that do not impact movement (e.g., Carr et al., 2008). Thus, our systems evolved in a natural environment with one set of contingencies, but sometimes operate in “artificial” environments where those contingencies no longer hold.

Although the processes that give rise to the GF have traditionally been thought to occur at a fairly high level of cognitive processing, the results reported here suggest that the locus of the GF might originate at a lower level of information processing. If this is the case our results have implications for the design of interventions intended to manage problem gambling. Specifically, they might imply that susceptibility to the GF is relatively impervious to learning and/or conscious control. This would in turn suggest that current intervention approaches (e.g., those that emphasize strategies such as reminding problem gamblers about the nature of randomness and laws of probability) may not be best. Rather, these intervention efforts might be better directed toward stressing the importance of taking breaks away from any string or run of gambling events. Given that, in even traditional cue-target situations, IOR effects can persist for several seconds (e.g., Tassinari et al., 1989; Samuel and Kat, 2003), a longer break would probably be better than a shorter break.

The results of these experiments also raise some intriguing questions for future study. For example, does the degree of response inhibition exhibited by one participant depend on the perceived competence of the other? Given that the results of our studies support the idea that there is a relation between the gamblers’s fallacy and IOR, the same type of question can be asked in a betting context. Specifically, do the betting behaviors and outcomes associated with a “competent” gambler have more or less impact on an observer’s behavior? Another future line of enquiry would be to apply these same protocols to an older population. Two things are well known: both the time-course and magnitude of IOR effects change with aging (e.g., McCrae and Abrams, 2001; Bao et al., 2004), and problem gambling behaviors can be particularly acute amongst the elderly (McNeilly and Burke, 2000). Typically, the reasons given for patterns of gambling behaviors in the elderly revolve around such sociocultural issues as depression and reduced levels of life satisfaction. Again, however, there may be more fundamental neurobiological factors that, at least in part, give rise to these behaviors. By expanding the original protocols developed here, it will be possible to determine if our predictions regarding the IOR-gambler’s fallacy relationship may help explain attitudes toward gambling in the elderly.

## Conflict of Interest Statement

The authors declare that the research was conducted in the absence of any commercial or financial relationships that could be construed as a potential conflict of interest.

## Footnotes

^1^Research on the perceived regulation of events has been driven, in large part, by Langer (1975) who demonstrated that people often behave as if chance events are accessible to personal control. Although Langer’s, 1975 work was one of the first to tackle issues of control illusions empirically, the genesis of the concept was likely formed in Goffman’s (1967) book *Interaction Ritual*.

^2^The apple example describes what one might call a “shift-to-win” situation. To be fair, there are also situations in which a shift in behavior has the potential to disadvantage the outcome. For example, when one catches a fish in a particular location it is normally advantageous to stick with that location, for at least a brief period of time, and to return to the location on subsequent fishing trips (i.e., a “stay-to-win” strategy). The point we make later in this paper is that, in the real world, people learn to make the most of non-random contingencies. These same contingencies do not apply in the random environment of a casino.

## Appendix

### Questionnaire

The following is a list of statements related to gambling. Please read each statement carefully and indicate how much you agree or disagree with it by circling the appropriate number. Please do not take too much time in responding to the items:

1. I consider myself to be a reasonably adept gambler.
  **Table:**
  1	2	3	4	5	6	7
  Don’t agree at all						Strongly agree
2. In some casinos, some gambling games (e.g., slot machines) are more likely to pay out than others.
  **Table:**
  1	2	3	4	5	6	7
  Don’t agree at all						Strongly agree
3. The longer a slot machine has gone without paying out a large sum of money, the more likely are the chances that that it will pay out in the very near future.
  **Table:**
  1	2	3	4	5	6	7
  Don’t agree at all						Strongly agree
4. It is a good idea to purposely avoid playing on slot machines that have recently paid out a lot of money.
  **Table:**
  1	2	3	4	5	6	7
  Don’t agree at all						Strongly agree
5. I believe that there is such a thing as winning and losing streaks.
  **Table:**
  1	2	3	4	5	6	7
  Don’t agree at all						Strongly agree
6. After a long string of wins on a slot machine, the chances of losing become greater.
  **Table:**
  1	2	3	4	5	6	7
  Don’t agree at all						Strongly agree
7. I am often willing to take risks.
  **Table:**
  1	2	3	4	5	6	7
  Don’t agree at all						Strongly agree
8. After a long string of losses on a slot machine, the chances of winning become greater.
  **Table:**
  1	2	3	4	5	6	7
  Don’t agree at all						Strongly agree
9. There is often a hidden pattern in most sequences of supposedly random events.
  **Table:**
  1	2	3	4	5	6	7
  Don’t agree at all						Strongly agree
10. A long string of wins or losses on a slot machine will have no influence on future wins or losses.
  **Table:**
  1	2	3	4	5	6	7
  Don’t agree at all						Strongly agree
11. If you ever experience a losing streak while gambling, the thought that a win has to coming soon should keep you going.
  **Table:**
  1	2	3	4	5	6	7
  Don’t agree at all						Strongly agree
12. If you toss a coin five times and the first four tosses come up heads, you think that the fifth toss will be more likely to be tails than heads.
  **Table:**
  1	2	3	4	5	6	7
  Don’t agree at all						Strongly agree
13. There are certain strategies that one should use when playing a slot machine (e.g., betting all remaining credits at once) that will help that person win.
  **Table:**
  1	2	3	4	5	6	7
  Don’t agree at all						Strongly agree
14. If others are winning in a gambling game, I feel that my turn is coming too.
  **Table:**
  1	2	3	4	5	6	7
  Don’t agree at all						Strongly agree
15. Sometimes, it is a good idea to keep gambling if you get a strong feeling that you are about to win.
  **Table:**
  1	2	3	4	5	6	7
  Don’t agree at all						Strongly agree
